# Residual Nitrite, Nitrate, and Volatile N-Nitrosamines in Organic and Conventional Ham and Salami Products

**DOI:** 10.3390/foods14010112

**Published:** 2025-01-03

**Authors:** Kathrine H. Bak, Susanne Bauer, Christoph Eisenreich, Peter Paulsen

**Affiliations:** 1 FFoQSI GmbH—Austrian Competence Centre for Feed and Food Quality, Safety and Innovation, Technopark 1C, 3430 Tulln, Austria; 2 Unit for Food Hygiene and Technology, Centre for Food Science and Veterinary Public Health, Clinical Department for Farm Animals and Food System Science, University of Veterinary Medicine Vienna, Veterinärplatz 1, 1210 Vienna, Austria; susanne.bauer@vetmeduni.ac.at (S.B.); christoph.eisenreich+public@gmail.com (C.E.); peter.paulsen@vetmeduni.ac.at (P.P.)

**Keywords:** cured meat, curing, human health, microbial safety, processed meat, volatile N-nitrosamines

## Abstract

Nitrite and nitrate in meat products may be perceived negatively by consumers. These compounds can react to form carcinogenic volatile N-nitrosamines. “Nitrite-free” (i.e., uncured) organic meat products may contain nitrate from natural sources (e.g., spices and water). We studied the quality of ham and salami (conventional cured; organic cured; organic uncured). Residual nitrite and nitrate, volatile N-nitrosamines, microbial load, surface color, water activity, and pH were determined, considering one week of refrigerated storage in open or unopened packages. Residual nitrite and nitrate in organic, uncured salami were similar to cured salami, presumably from the addition of herbs and spices and nitrate reduction by nitrate reductase from microorganisms. For cooked ham, residual nitrite was significantly lower in the organic, uncured sample, while residual nitrate was not detected. N-nitrosodiphenylamine was detected in all samples at day 0, exceeding, in three out of five cured and both uncured products, the US legal limit of 10 µg/kg of volatile N-nitrosamines in foods. This finding warrants further investigation. The microbial load in salami products was dominated by bacteria from starter cultures. In ham, a slight increase in total aerobic count and lactic acid bacteria during storage was noted. Overall, the microbial quality of the products was as expected for the respective product types.

## 1. Introduction

Currently, there is an increasing consumer demand for more so-called “clean label” products [1]. Clean label is a vague, non-scientific term used to describe food products that are made with as few ingredients as possible and with no added chemicals or artificial ingredients [2]. Health-conscious consumers are more likely to purchase organic food products, which are perceived as more clean label, healthier, and environmentally friendly than conventional foods [2,3,4,5]. For meat products, specifically, health-conscious consumers tend to choose products with a lower salt and nitrite contents [6]. This trend drives the industry’s desire for the removal of nitrite and nitrate from meat products. For example, many types of organic meat products are produced without added nitrite/nitrate, which is particularly evident from the Danish ban on nitrite and nitrate in organic meat products, although it is allowed in the rest of the EU [7].

Curing meat with nitrite or nitrate provides microbiological safety in addition to the anticipated cured meat color and flavor [8,9]. The effective ingredient is nitrite, but nitrate can be reduced to nitrite by certain microorganisms added to raw meat products [10]. However, the typical cured color and flavor can be obtained from food ingredients other than nitrite and nitrate. For instance, the nitrate content of vegetables and drinking water is relatively high [1]. Celery powder is an example of an ingredient, which can be used together with starter cultures to produce what some term “naturally cured” meat products [11]. The curing reaction between nitrite and myoglobin proceeds similarly no matter the origin of the nitrite, though, the risks of batch-to-batch variation and sub-optimal curing are greater when nitrite originates from nitrate-containing ingredients due to the variability in the amount of nitrite formed [12].

Food additives, including the amount of nitrite and nitrate allowed in cured meat products, are regulated in the EU by Regulation (EC) No 1333/2008 [13] and Commission Regulation (EU) 2023/2108 [13,14]. The legal amounts are based on the expert opinion of the European Food Safety Authority (EFSA) to keep the level of N-nitrosamines in cured meat products as low as possible while maintaining the microbiological safety of the products, particularly the inhibition of *Clostridium botulinum* [13]. The amounts of sodium nitrite (E 250), potassium nitrite (E 249), sodium nitrate (E 252), and potassium nitrate (E 251) are given as either the maximum amount that may be added during production or as the maximum residual amount (expressed as NaNO_2_), depending on the product type [13]. Generally, 150 mg/kg of NaNO_2_ may be added during the production of cured meat products [14,15]. It is worth mentioning that EU Commission Decision 2024/1225 [16] allows Denmark to have special regulations, meaning that only 60 mg/kg of NaNO_2_ is allowed when producing cured meat products in Denmark. Furthermore, according to Danish law, organic meat products produced in Denmark cannot be cured using nitrite salt [17] unless an exemption is granted, which has not yet been achieved [7,18].

Nitrite may react to form a nitrosating agent, which can react with secondary amines to form N-nitrosamines, especially under acidic conditions and as a result of heat treatment or storage [10,19]. Many N-nitrosamines (especially the volatile N-nitrosamines) are carcinogenic [19,20] by causing DNA damage [21]. Epidemiological studies have shown a positive correlation between the intake of processed meats containing volatile N-nitrosamines and the occurrence of more than one type of cancer [22]. Despite this, not many countries have legal limits for the level of N-nitrosamines in food. No limit has been established in the EU as of yet [19], but EFSA recently determined a lower confidence limit of the benchmark dose at 10% to be 10 µg/kg body weight per day for the ten known carcinogenic volatile N-nitrosamines found in food (N-nitrosodimethylamine (NDMA), N-nitrosomethylethylamine (NMEA), N-nitrosodiethylamine (NDEA), N-nitrosopyrrolidine (NPYR), N-nitrosomorpholine (NMOR), N-nitroso-di-n-propylamine (NDPA), N-nitrosopiperidine (NPIP), N-nitrosodi-n-butylamine (NDBA), N-nitrosodiphenylamine (NDPhA), and N-nitrososarcosine (NSAR)) [23]. On the other hand, a legal limit of 10 µg volatile N-nitrosamines/kg food was set in the United States [19,20], a limit which European meat products generally fall below [24]. Studies investigating the dietary exposure and risk assessment from the intake of volatile N-nitrosamines due to the consumption of processed meat products have found that this exposure is of low concern [22,25]. However, a negative effect cannot be ruled out due to the aforementioned genotoxic effect of volatile N-nitrosamines [22].

To the best of our knowledge, no studies have been conducted looking specifically at the contents of residual nitrite, nitrate, and volatile N-nitrosamines in commercial organic cured and uncured meat products. Thus, the levels of residual nitrite, nitrate, and nine of the carcinogenic volatile N-nitrosamines (NDMA, NMEA, NDEA, NPYR, NMOR, NDPA, NPIP, NDBA, and NDPhA) were investigated in organic cured and uncured ham and salami products before and after a week of refrigerated storage with conventional, cured varieties of the products serving as controls. Similarly, the surface color, indicative of cured meat color formation, was measured along with water activity (a_w_) and pH. Additionally, total aerobic mesophilic bacteria, Enterobacteriaceae, *Pseudomonas*, lactic acid bacteria (LAB), and Staphylococci were determined before and after a week of refrigerated storage to establish the microbial quality of the meat products.

## 2. Materials and Methods

### 2.1. Sample Selection

A market assessment was conducted by visiting all supermarket chains available in Vienna as well as searching Austrian web shops for cooked deli ham and salami products from each of the following three groups: (1) organic-traditionally cured (ham: HOC1 and HOC2; salami: SOC), (2) conventional-traditionally cured (reference/control; ham: HCC; salami: SCC), and (3) organic-uncured (ham: HOU; salami: SOU). No organic, uncured, cooked ham was found in Vienna/Austria, so this product was purchased from Denmark. Organic food products are defined according to EU regulation (EU) 2018/848 as being manufactured from ingredients from organic production [26]. The product groups are displayed in Table 1.

The samples purchased in supermarkets in Vienna were immediately transported to the University of Veterinary Medicine Vienna and kept refrigerated (4 °C) until the analyses were initiated (either on the day of purchase or the following day). Ten packages (replicas) of each product were purchased, except for HOC1 where only three packages were available. Sample HOC2 was purchased as an additional representative of organic, traditionally cured ham.

Sample HOU was purchased in a Danish supermarket and stored refrigerated (5 °C) until transport to Vienna, Austria. Transportation was carried out by car (approx. 11 h) with the samples stored in an insulated cooling bag containing several cooling blocks. The temperature was checked upon arrival in Vienna and remained at <5 °C.

Samples purchased from the internet were shipped in an insulated box packed with Styropor foam, picked up from the post office at the University of Veterinary Medicine Vienna, and kept refrigerated (4 °C) until the analyses were initiated.

The samples were analyzed before (day 0) and after one week (day 7) of refrigerated storage (4 °C in the dark) in opened or unopened packages according to the experimental plan shown in Table 2. The end of storage (day 7) was always on or before the best-before date.

### 2.2. Physicochemical Analyses

#### 2.2.1. Determination of Nitrite Content

The amount of residual nitrite as NaNO_2_ was analyzed enzymatically according to DIN EN 12014-3 [27]. In brief, the sample was homogenized with H_2_O, brought to pH 8–8.5 and, after boiling for 15 min, clarified with Carrez solution and filtered. A color reagent (sulfanilamide/N-(1-naphthyl)-ethylenediammonium dichloride) was added to the filtrate and measured photometrically at 540 nm after 30 min of incubation. The limit of detection (LOD) was 1.4 mg NaNO_2_/kg.

#### 2.2.2. Determination of Nitrate Content

The samples were prepared as described in Section 2.2.1, and then filtered through a 0.2 µm membrane filter prior to analysis. The content of residual nitrate measured as KNO_3_ was analyzed via high-performance liquid chromatography (HPLC) by a method adapted from DIN EN 12014-2 and Schmidt and Schwedt [28,29] with the modification that the column used was a Spherisorb Amino (NH_2_) Column, 80 Å, 5 μm, 4.6 mm × 250 mm. The mobile phase consisted of a 95% KOH buffer (pH 3) and 5% acetonitrile (isocratic, 0.5 mL/min). Detection was carried out using a PDA detector at 205 nm. A KNO_3_ solution served as a standard. The LOD was 0.1 mg KNO_3_/kg.

#### 2.2.3. Determination of N-Nitrosamines Content

The content of nine volatile N-nitrosamines was analyzed via gas chromatography–mass spectrometry (GC-MS) according to Roasa et al. [30] using an Agilent 8890/5977B GC/MSD (Agilent Technologies, Vienna, Austria). The nine volatile N-nitrosamines measured were NDMA, NMEA, NDEA, NPYR, NMOR, NDPA, NPIP, NDBA, and NDPhA. These comprise nine of the ten carcinogenic volatile N-nitrosamines considered by EFSA, the tenth being NSAR [23].

Approximately 1 g of chopped meat was weighed into 20 mL GC vials and stored at −80 °C until analysis. For the analysis, the samples were mixed with 3 mL of H_2_O, shaken, and then extracted onto an SPME fiber DVB/CAR/PDMS 50/30 µm (Agilent Technologies, Vienna, Austria) at 65 °C for 45 min. The GC-MS run was carried out in splitless mode with helium as the carrier gas. The oven temperature started at 80 °C for 2 min then increased to 100 °C at a rate of 10 °C/min and to 120 °C at a rate of 2 °C/min and was held at 120 °C for 12 min. The temperature was increased to 280 °C at a rate of 20 °C/min and was held for 4 min. The LODs for the nine volatile N-nitrosamines were as described by Roasa et al. [30] and ranged from 0.16 µg/kg for NDEA to 56 µg/kg NDMA, while the LOD for NDPhA was 16 µg/kg. The limits of quantification were a little over three times higher than the LODs [30]. The concentration of volatile N-nitrosamines in the meat samples was calculated based on a standard curve. The standard curve (0–1000 µg N-nitrosamine/mL) was prepared by diluting the 2000 µg/mL standard mixture (EPA 8270/Appendix IX Nitrosamines Mix; Merck, Darmstadt, Germany).

#### 2.2.4. Measurement of Surface Color

Surface color was measured on day 0 and day 7 of storage. The measurements were carried out using a handheld Nix QC Color Sensor—MS (Nix Sensor Ltd., Hamilton, ON, Canada). The device was calibrated according to the manufacturer’s instructions before measuring surface color as lightness (L*), redness (a*), and yellowness (b*) using illuminant D_65_, 14 mm aperture size, and a 10° observer angle. Measurements were conducted on the top slice of the package and according to guidelines to avoid any influence of sample translucency [31,32]. The results were presented as an average of triplicate measurements of three packages of each product: (1) on day 0 before storage of the samples in opened packages; (2) on day 7 after storage of the samples in opened packages (same samples as day 0); and (3) on day 7 after storage of the unopened samples. The only exception was sample HOC1 where only one package was measured (i.e., triplicate measurements on one package) in each of the three instances.

#### 2.2.5. Determination of Water Activity

The water activity of the samples was determined according to ISO 18787:2017 [33] via measurement of relative humidity (Lab-Swift, Novasina, Lachen, Switzerland). The results are given as the average result for three packages per sample, except for sample HOC1 where ham from only one package was measured at each time point.

#### 2.2.6. Measurement of pH

The pH of the samples was measured with a pH meter (Testo 230, Testo, Titisee, Germany, with a SI Analytics™ BlueLine pH combination electrode, Schott Instruments, Mainz, Germany). The pH meter was calibrated with buffers at pH 7 and pH 4 before sample measurement. Results are given as the average result for three packages per sample, except for sample HOC1 where ham from only one package was measured at each time point.

### 2.3. Microbiological Analyses

The microbiological examination was performed via cultural methods and included the enumeration of total aerobic mesophilic bacteria (Plate Count Agar; incubation three days at 30 °C) according to ISO 4833-1:2013 [34]; Enterobacteriaceae (VRBG agar; incubation one day aerobic at 37 °C) according to ISO 21528-2:2017 [35]; *Pseudomonas* (GSP-agar; incubation three days at 25 °C) [36], and LAB (MRS Agar; incubation two days at 37 °C) according to ISO 15214:1998 [37]. Staphylococci were enumerated on Baird-Parker Agar with egg yolk supplement (incubation two days at 37 °C) (Merck, Darmstadt, Germany). Characteristic black colonies with turbid halos (degradation of lecithin) were considered presumptive *Staphylococcus aureus*.

Sample preparation for enumeration of bacteria was as follows: From the sample units, a total of 25 g was taken and mixed with 225 g Maximum Recovery Diluent (MRD) in a Stomacher lab Blender 400 to give a suspension 1:10. Serial tenfold dilutions were prepared in MRD, and aliquots of 0.1 mL were spread onto the respective agar plates. After incubation, the number of characteristic colonies was counted, and the number of colony-forming units (cfu)/g was calculated. The lower limit of detection (LLOD) was 100 cfu/g. Results < LLOD were given the conservative (in terms of microbiological safety) value “99”. Results were then log transformed to give log cfu/g.

### 2.4. Statistical Analysis

Analysis of variance (ANOVA) for each product type (salami and cooked ham) for the parameters a_w_, pH, and content of nitrite, nitrate, and N-nitrosamines was carried out in Excel (Microsoft 365 version). *p*-values ≤ 0.05 were considered significant. Post hoc tests were performed when *p* ≤ 0.05. Tukey’s test was performed to conduct multiple comparisons when sample sizes were equal (https://astatsa.com/OneWay_Anova_with_TukeyHSD/, accessed on 10 October 2024, whereas Dunn’s test was used when sample sizes were not equal, i.e., for cooked ham (https://www.statskingdom.com/kruskal-wallis-calculator.html, accessed on 10 October 2024). For residual nitrite and nitrate as well as for N-nitrosamines, sample HOC1 was left out of the post hoc analysis, and Tukey’s test was performed on the remaining samples.

Color before and after storage of the same opened samples was compared using a paired *t*-test (Excel, Microsoft 365 version), whereas color after storage of the samples stored opened and unopened, respectively, was compared using an unpaired *t*-test (Excel, Microsoft 365 version).

Microbial counts were converted into log values and two-way ANOVA with Tukey’s post hoc test to discriminate among means. In sample sets with unequal variances, the Kruskal–Wallis test was used (StatGraphics 3.0; Statgraphics Technologies Inc., The Plains, VA, USA).

The results are represented by arithmetic means with their 95% confidence intervals (corrected for small sample sizes [38]) to visualize the order of magnitude of differences.

## 3. Results and Discussion

**Figure 1:**
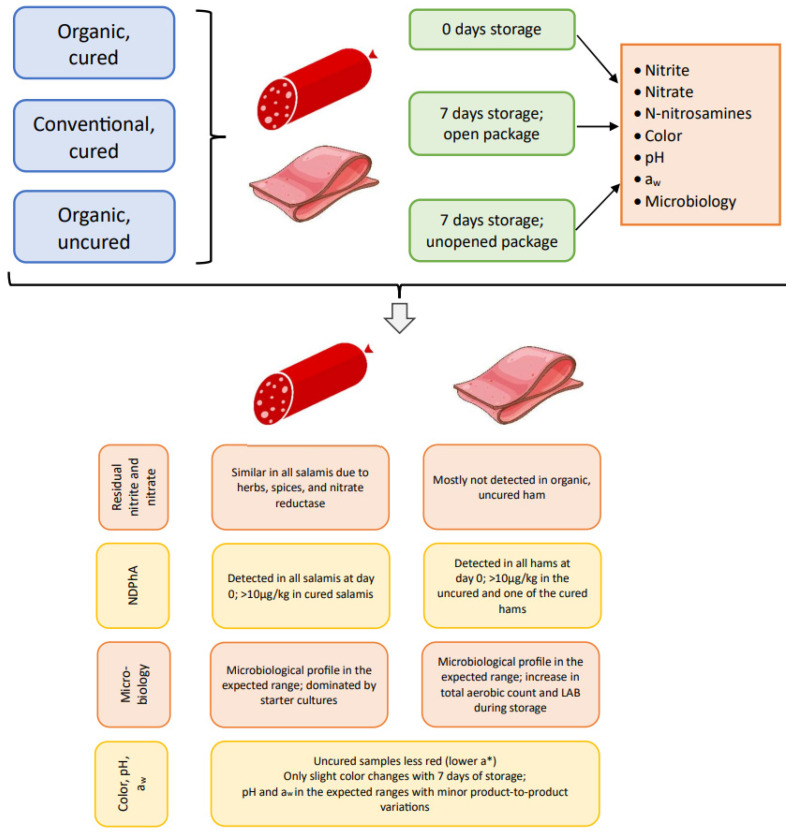
An overview of the experimental design and a summary of the main findings.

The results of all analyses are presented and discussed below. An overview of the most significant results is presented in Figure 1.

### 3.1. Physicochemical Traits

#### 3.1.1. Nitrite and Nitrate

All samples, except sample HOU, contained residual nitrite and nitrate, regardless of storage conditions (Table 3).

For the salami products, nitrite was highest in sample SOC and, not surprisingly, lowest in sample SOU, though, the difference was only statistically significant on day 7 after unopened storage. Nitrite and nitrate in an uncured product may originate, in part, from the nitrite and nitrate present in the raw pork from nitrogen metabolism and feed [39,40]. In addition to drinking water and vegetables [1], herbs and spices are known to contain nitrate [41,42], which can be reduced to nitrite via nitrate reductase produced by bacteria present in the fermentation flora [43]. The uncured salami product in question (SOU) contained 7% Mediterranean herbs in addition to spices, which may well explain the lack of difference in residual nitrate content between SOU and SOC (Table 3). Residual nitrate was highest in sample SCC, but the difference was only significant on day 0, as the level of residual nitrate declined (though, not statistically significant) from day 0 to day 7 likely due to nitrate reductase from the starter cultures. The high residual nitrate content in sample SCC likely originated from the added potassium nitrate, beetroot juice concentrate, and spice extracts that were among the ingredients.

For the ham products, measurements for residual nitrite for sample HOU (the organic, uncured product purchased in Denmark) were below LOD on day 0 as well as day 7 in the opened sample, whereas a small amount of nitrite was detected in two out of three replicas of the sample stored unopened. As this product contained no spices, the detected nitrite must originate either from the meat itself or from the small amount of added water. Residual nitrate was below LOD for sample HOU. For the cured ham samples, there was a small, but significant, difference in residual nitrite content between one of the organic, cured products (HOC2) and the conventional product (HCC) (Table 3). Other authors have reported a decrease in nitrite content during the storage of sausages [44,45], which is not consistent with the current results. However, this may be due to the differences in storage time, which was one week in our case, while the other studies applied four weeks [44] and ninety days [45], respectively. In the latter study, the first measurement was made after two weeks where the decrease in residual nitrite was not significant for all treatments [45].

Residual nitrate content in sample HOC2 on day 7 of storage was significantly higher than the residual nitrate content in all other ham samples (Table 3). In sample HCC, residual nitrate content remained constant during storage (Table 3). This finding is in line with previous reports [45]. A constant level of residual nitrate during storage is likely due to redox reactions causing residual nitrite to be converted to nitrate, which then remains constant even with a prolonged storage period [45].

The levels of nitrite and nitrate added to the products are not known. However, the results indicate that these were below the EU legal limits. A previous study analyzed the nitrite content in various types of meat products from the time of production and at several time points until the use-by date (from 28 to 56 days after production). The nitrite content expressed as mg NaNO_2_ per kg ranged from approx. 105–120 mg/kg at the time of production to approx. 5–52 mg/kg on the respective use-by dates [15]. The residual nitrite levels for our samples fall within this range, and it would be plausible to assume that the levels of added nitrite were thus below the EU legal limit for all samples. A residual nitrate level above the EU legal limit of 150 mg NO_3_^−^/kg was found for 10 out of 300 analyzed meat samples in a different study, ranging from 230.9 ± 55.4 mg/kg to 2575.2 ± 618.0 mg/kg [46]. The residual nitrate level in all samples analyzed in our study is well below the levels found in the non-compliant samples [46].

Consumers should be made aware that choosing an organic meat product does not necessarily eliminate the intake of residual nitrite and nitrate since organic meat products are cured in most EU countries, and even uncured meat products may contain residual nitrite and nitrate due to nitrate-containing ingredients in the recipe.

#### 3.1.2. Contents of N-Nitrosamines

N-nitrosamines are characterized by a nitroso group to an amine, and around 300 congeners are known today. Although the mode of formation was described in the second half of the 19th century, adverse health effects of this group of compounds were reported in the 1950s [47]. Short-term health effects are genotoxicity and acute oral toxicity, while N-nitrosamines can cause cancer in various organs as long-term effects [48], but the correct estimation of the mutagenic and carcinogenic potency is currently possible only for select N-nitrosamines [48].

The pathways of exposure of humans to N-nitrosamines include not only ingestion of food, beverages, and water, but also tobacco and personal care products [49]. In water, N-nitrosamines can occur as by-products of water disinfection [50] and contribute directly or indirectly—via agri- and aquacultural products—to human exposure.

Gushgari and Halden [49] critically reviewed the existing literature on human exposure to N-nitrosamines. Expectedly, consumption of tobacco products results in significant N-nitrosamines exposure for consumers. With respect to nutrition, the consumption of alcoholic beverages, but also of meat and fish (and products thereof) is of concern. Arguably, a change in lifestyle (i.e., to avoid tobacco and alcoholic beverages), and a shift from meat protein to plant protein, e.g., tofu, will result in reduced exposure for consumers [49]. The authors estimated that, in the US, 600 cases of cancer per 100,000 inhabitants are attributable to N-nitrosamines, but 92% of these cases could be avoided by the abovementioned lifestyle changes.

Mitigation strategies may include not only changes in lifestyle, but also an array of food technology measures, which are available to produce foods with low N-nitrosamine content [51]. The reduction in N-nitrosamines in beer is a documented story of success (achieved by indirect firing during malt roasting) [52], but also for cured meats, a number of mitigation strategies exists, including the following examples: (a) keeping content of precursors (i.e., nitrites, nitrates, and biogenic amines) in raw materials low; (b) avoiding direct exposure to fire during heat processing; and (c) using certain (antioxidant) additives or inactivating N-nitrosamines by gamma irradiation [51]. Current EU legislation aims to reduce nitrite and nitrate levels in foods [13,14], and member states can release more strict regulations, as done by Denmark [16,17].

The only volatile N-nitrosamine we detected was NDPhA. This N-nitrosamine was found in all ten samples on day 0 (Table 4), but not in the stored samples. NDPhA belongs to the group of possibly carcinogenic compounds, group 2B, as classified by the International Agency for Research on Cancer (IARC) [53,54]. The content of NDPhA in salami SOC was significantly higher than in sample SCC and sample SOU. The content of NDPhA in cooked ham HOC2 was significantly lower than in sample HCC, whereas there was no difference between sample HOU and either sample HOC2 or sample HCC. Some studies suggest that a higher fat content in the meat product results in an increased formation of volatile N-nitrosamines [55], while others have found no significant effect of fat content on their formation [56]. While we did not statistically compare the levels of NDPhA between product types, it would appear that the ham samples (2–10 g fat/100 g) on average have a slightly higher content of NDPhA than the salami samples (34–39 g fat/100 g), though with rather large standard deviations. Interestingly, even the nitrite-free samples (SOU and HOU) contained NDPhA, which is especially surprising for sample HOU, which contained basically no residual nitrite or nitrate (Table 3).

NDPhA was detected as a disinfectant by-product in drinking water formed from the chloramination of the precursor DPhA [57]. Another route of formation in water is via nitrosation of DPhA [58], which could potentially also take place in cured meat.

Our results for volatile N-nitrosamines are mostly surprising when compared to the majority of the literature. The content of N-nitrosamines in meat products was reviewed by Flores et al. [24], who state that NDMA, NDEA, NPIP, NPYR, and NMOR are the most commonly detected N-nitrosamines in meat products. Furthermore, NDPhA was not detected in four different processed meat and poultry products, which the authors attributed to the lower sensitivity of the applied method for this particular N-nitrosamine [30]. Likewise, in a different study, the NDPhA content was below the limit of quantification in five different processed meat products both on the day of purchase and after seven days of refrigerated storage [20]. On the other hand, NDPhA was one of the main N-nitrosamines detected in a study investigating the N-nitrosamine content of numerous different processed meat and fish products [59]. However, NDPhA was detected at lower concentrations (maximum 20 µg/kg) [59] than what we detected in many of our samples (Table 4). NDPhA was also detected in a study [60] where the effect of three natural antioxidants on the formation of N-nitrosamines in smoked sausage was investigated. Generally, the addition of natural antioxidants led to a reduction in the formation of N-nitrosamines, except for NDPhA, which increased with the addition of grape seed extract and green tea polyphenols, while the content of NDPhA was reduced with the addition of rosemary extract [51,60]. However, NDPhA contents were in the range of approx. 200 to approx. 5500 µg NDPhA/kg for the smoked sausage [60], which is higher than the results in our study (Table 4). Further studies on Austrian cured meat products seem advisable, as the current standing shows that on day 0, three out of five cured and both uncured products have contents of NDPhA exceeding the US legal limit of 10 µg/kg of volatile N-nitrosamines in cured meat.

Generally, volatile N-nitrosamines do not appear to be a big problem in European meat products. The content of volatile N-nitrosamines in processed meat products in several European countries was below 10 µg/kg [24]. In a Danish study, volatile N-nitrosamines of 100 processed meat products were found to be in the range 0.1–7.7 µg/kg, except for one bacon sample, which contained 11.5 µg/kg due to 10.8 µg/kg of NPYR [61], highlighting the rarity of a high content of volatile N-nitrosamines in cured meat products on the European market. Nevertheless, Bonifacie et al. [62] raise the important point that the effects of a second cooking step must be considered (e.g., pizza toppings), which is in line with the statement that N-nitrosamine formation happens primarily at temperatures above 130–140 °C [10,43,63]. It should be noted that volatile N-nitrosamines can also be formed in the acidic environment of the stomach from ingested nitrite and nitrate [19] and may thus stem from other sources than cured meat. On the other hand, components such as lipid oxidation products and heme present in meat and meat products are also carcinogenic [21].

Bioactive compounds were found to reduce the production of some volatile N-nitrosamines [53], though some antioxidants may increase the production of certain volatile N-nitrosamines, as previously discussed [60]. If the bioactive compounds originate from a nitrate-containing source, the production of other volatile N-nitrosamines may increase due to nitrosation [53]. Specifically, black pepper and paprika were linked to the formation of NPIP and NPYR [64,65,66]. The products used in this study generally contain undisclosed spices, though samples SOC and HCC also contain garlic while sample HOU contains no spices at all but does contain an antioxidant. Sample SOU contains smoke. Smoked meat was shown to contain an increased amount of volatile N-nitrosamines [55,61], though NDPhA was not mentioned as one of the N-nitrosamines that increase with smoking.

#### 3.1.3. Water Activity and pH

Among the salami samples, the organic, cured variety (SOC) had the lowest water activity at day 0. In closed packages stored for 7 days, no statistically significant change in a_w_ was noted, whereas a_w_ significantly declined in the packages stored open for 7 days. This was most probably because of the drying of the sausage slices (Table 5).

Conversely, SOC demonstrated the highest pH at day 0, which could be due to minor processing differences, utilizing the hurdle technology slightly differently [67] or simply because of different fermentation and drying periods for the three salamis (production specifics unknown). The differences in pH between the three products remained during storage, and pH did not change significantly with storage (exception: SOC, day 7, open).

In cooked ham samples, a_w_ differed significantly between samples at day 0. During storage, a_w_ significantly declined in HOC2, while a_w_ remained stable in samples HCC and HOU. The pH of the ham was significantly higher in HCC than in HOC2 and HOU. During storage, pH declined, though not always significantly (Table 5). This might be explained by acid formation by LAB during storage (see Section 3.2.4).

#### 3.1.4. Surface Color

No significant color differences occurred for the salami products from day 0 to day 7 of storage in opened packages (Table 6). However, a* was slightly higher on day 7 of unopened storage than storage in open packages for samples SOU and SCC, i.e., color fading during storage in open packages. The combination of light and O_2_ is what causes the photodissociation of NO from the cured pigment, resulting in color fading [32,43]. Numerically, a* values were lowest in sample SOU, which is not surprising, since this was the uncured product, and thus had the least amount of residual nitrite and nitrate (Table 3). Nitrite is essential for the formation of the characteristic red/pink cured meat color characteristic of the pigment nitrosylhemochrome [32,43].

For cooked ham, there were no differences in a* due to storage time and conditions (Table 6), indicating that no photodissociation took place during storage. The conventional, cured ham (HCC) became darker (lower L*) and less yellow (lower b*) with storage in an open package, whereas the organic, uncured ham (HOU) became more yellow (higher b*) with storage in an open package. Lower b* values were seen with storage in unopened compared to open packages for samples HOC2 and HOU (Table 6).

Even though the color variables were not compared statistically between products, it is evident that the uncured products SOU and HOU are less red (lower a*) than their cured counterparts. This is not surprising, since HOU has no detectable residual nitrate and only a small amount of residual nitrite at day 7 after storage in unopened packages (Table 3), resulting in only a negligible degree of cured color formation. Although the levels of nitrite and nitrate in SOU were not statistically different from both of the cured salamis (SOC and SCC), the amounts detected were numerically lower, which could explain the lower a* value.

### 3.2. Microbiological Analyses

As previously mentioned, the LLOD was 100 cfu/g. Hence, in cases where the microbial count was <100 cfu/g, 99 cfu/g was used for the log transformation as a conservative estimate to err on the side of caution. This means that bars with a height of “2” and a standard deviation of “0” indicate that results were below the LLOD.

#### 3.2.1. Total Aerobic Count

**Figure 2:**
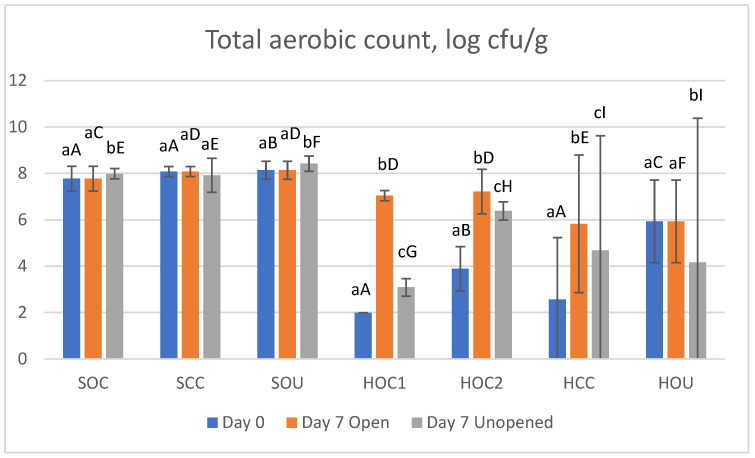
Total aerobic count in studied meat products; data are log cfu/g. Bars represent mean values, and 95% confidence intervals are indicated by error bars. Reference Table 1 for sample IDs. For each product, different lowercase letters above bars indicate statistically significant differences between storage conditions, whereas different capital letters indicate statistically significant differences between different salami or ham products at same storage condition (*p* ≤ 0.05).

The total aerobic count for all samples is shown in Figure 2 (reference Table 1 for sample IDs).

The total aerobic count was similar in all salamis at both day 0 and day 7 (open and unopened storage). Statistically significant differences were noted, albeit in the order of magnitude of merely 0.3 log cycles. These counts are expected in fermented products due to the abundance of Gram-positive bacteria in the starter cultures [68].

The ham products saw higher total aerobic counts on day 7, particularly in the samples that were stored in open packages, though not all differences were significant. In contrast to fermented products, cooked hams are pasteurized during manufacturing, which reduces the total bacterial counts, but does not effectuate sterilization. These products have a limited shelf life under refrigerated conditions and a rise in bacterial numbers during (cold) storage is expected [67].

Since nitrites/nitrates exert an antibacterial effect, which differs between species [69], it could be expected that total aerobic counts are higher in the uncured sample. Although this held true for the initial counts (day 0) in hams, the (organic) uncured ham (HOU) demonstrated no changes after seven days of open storage. Since this product had the lowest pH of all the studied hams, this might have contributed to the microbiological stability of the product.

#### 3.2.2. Enterobacteriaceae

The Enterobacteriaceae counts were consistently <LLOD in all samples from the salami varieties and in hams HOC1, HOC2, and HCC. In ham sample HOU, Enterobacteriaceae counts were on average < 2 log cfu/g in the samples tested at day 0 and day 7 (open package). In one of the three HOU samples stored for 7 days in unopened packages, the average Enterobacteriaceae count was 2.8 log cfu/g, which results in a rather high standard deviation of the average result.

In fermented meat products, Enterobacteriaceae are eliminated during the acidification and decline of the redox potential caused by the growth and/or metabolic activity of the facultative anaerobic *Lactobacillus* present in the starter culture [67]. In cooked meat products, Enterobacteriaceae are readily inactivated by heat [70]. In cooked, cured meat, residual nitrite provides a small hurdle against the growth of certain bacteria [43], and sample HOU contains basically no residual nitrite (Table 3). The increase in Enterobacteriaceae in a single unopened packing unit of HOU may be attributable to some contamination before or during packaging. Enterobacteriaceae generally cause spoilage of meat products and can be indicative of defects or contamination events in meat processing [70].

#### 3.2.3. Pseudomonas

The *Pseudomonas* counts in the three salami varieties were below LLOD. Like Enterobacteriaceae, this was expected since Gram-negative bacteria are readily eliminated during the ripening phase in salami manufacturing [67].

*Pseudomonas* were present above LLOD in a few packages of the ham samples. Sample HOC2 had an elevated count on day 7 of opened storage (2.91 ± 1.07 log cfu/g), and the same was the case for sample HCC, though with a lower count (2.17 ± 0.32 log cfu/g). Since aerobic storage at low temperatures favors the growth of *Pseudomonas*, this finding was not unexpected [61]. Detectable levels of strictly aerobic *Pseudomonas* in HOU stored for 7 days in unopened packages (2.28 ± 0.67) were unexpected but could indicate some contamination before sealing the package or some defect of the package itself. Notably, this product/storage combination also exhibited higher numbers of Enterobacteriaceae.

#### 3.2.4. Lactic Acid Bacteria

**Figure 3:**
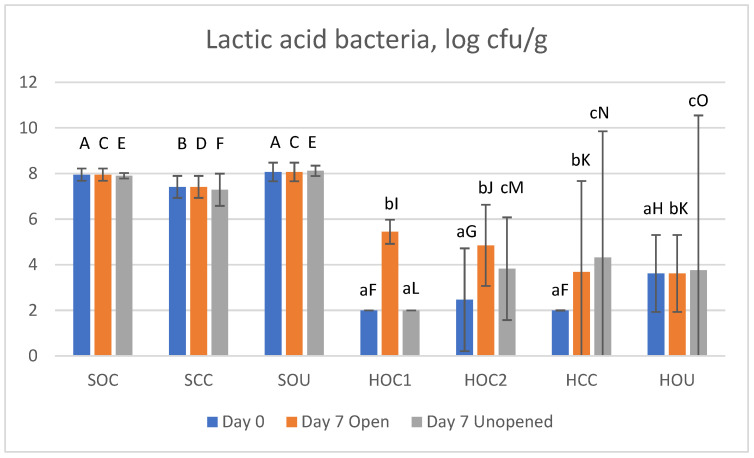
Lactic acid bacteria count in studied meat products; data are log cfu/g. Bars represent mean values, and 95% confidence intervals are indicated by error bars. Reference Table 1 for sample IDs. For each product, different lowercase letters above bars indicate statistically significant differences between storage conditions, whereas different capital letters indicate statistically significant differences between different salami or ham products at same storage condition (*p* ≤ 0.05).

The LAB count in the samples is shown in Figure 3 (reference Table 1 for sample IDs). The count was naturally high in all three salami products due to their application in starter cultures [68]. The difference between salamis SOC, SCC, and SOU and the different storage times/conditions were minimal. Admittedly, the cured product SCC demonstrated significantly lower LAB counts, yet in the order of merely 0.5 log cycles. For the ham samples, the LAB count was below the limit of detection for sample HOC1 on day 0 and day 7 of unopened storage as well as for sample HCC on day 0. Since ham is generally mildly heated, it is not unexpected that some thermotolerant LAB may survive [63]. Likewise, the slicing process can cause some microbiological contamination. Finally, the anaerobic milieu in the package can favor the growth of LAB. The results show some significant differences, yet no clear pattern regarding the mode of storage or the use of curing agent since LAB can actually degrade nitrite [71].

Interestingly, the (organic) uncured ham (HOU) demonstrated similar LAB counts irrespective of storage condition. The comparably low initial pH may have accounted for that.

#### 3.2.5. Staphylococci

**Figure 4:**
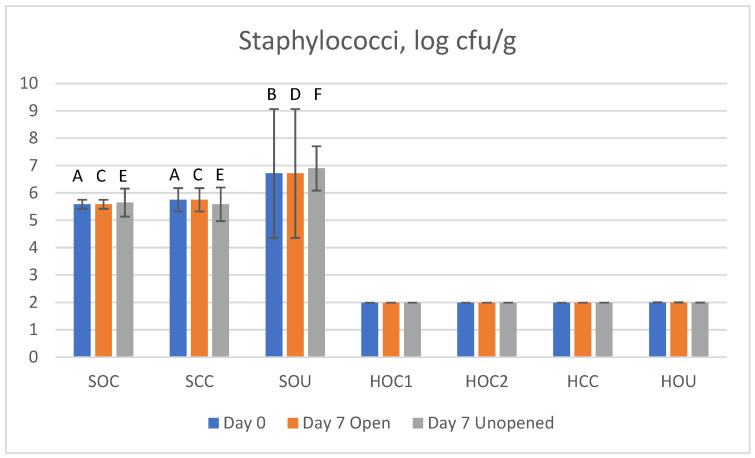
Staphylococci count in studied meat products; data are log cfu/g. Bars represent mean values, and 95% confidence intervals are indicated by error bars. Reference Table 1 for sample IDs. For each product, different capital letters indicate statistically significant differences between different salami or ham products at same storage condition (*p* ≤ 0.05).

The Staphylococci count for all samples is shown in Figure 4 (reference Table 1 for sample IDs). Colonies with a turbid halo indicative of lecithinase activity were not detected. Staphylococci were detected in all salami samples under all storage conditions, which is not surprising since they are part of the starter culture used for the production of salami [68]. A statistically significant higher number was noted in the uncured product (SOU) compared to the cured products (SOC and SCC). Since details on ingredients (starter cultures) and ripening conditions were not available, it remains to be elucidated in further studies if this finding was associated with the presence/absence of a curing agent. On the other hand, Staphylococci were below the LLOD for the ham samples, which is not surprising for pasteurized products.

### 3.3. Consumer Perspectives and Clean Label Trends

Health-conscious consumers are more likely to buy organic food products; environmental consciousness and sustainability also play a large role [3,4,5,72]. Specifically, environmental consciousness and sustainability appear to be important reasons for organic meat purchase [73]. On the other hand, reasons that were listed by consumers against purchasing organic meat include, for organic lamb, the lack of supply as the main factor, followed by the higher price and a lack of trust in the organic labeling; the belief that organic is not better than conventional was also of importance, but only to a minor degree [74]. It is likely that these factors can be transferred to organic meat from other animals along with organic meat products, though supply is possibly less of an issue with more common meat types such as beef and pork. Even though organic meat is more expensive, studies show that many consumers are willing to pay a premium, for example, for organic beef [75], although the price is also one of the main purchasing barriers [73].

Organic foods fall under what the consumers perceive as “clean label” products [76]. Although “free from artificial additives/ingredients” also falls under the clean label umbrella, it should be noted that it is not necessarily the same as organic [76], which is also evident from the occurrence of organic, cured meat products. A recent review on clean label in the meat industry drew attention to the fact that what consumers consider to be “clean label” is based on their perception. For example, ingredients that are difficult to pronounce are generally not considered to adhere to the clean label strategy even if they are natural [77]. It is further emphasized that the lack of communication from the meat industry adds to the confusion among consumers. This includes the use of natural curing ingredients and how they chemically react the same way in meat curing as synthetic nitrite and nitrate [77]. Another review on the same topic also highlights the importance of the meat industry educating the consumers as well as the need for the “clean label” term to be legally defined by the appropriate authorities [78].

As an example, an Italian study on the willingness to buy salami products with different characteristics (price; normal vs. reduced salt; presence or absence of nitrite; national origin) found that the most important characteristics for perceived salami quality were price (high), salt content (reduced), and nitrite (absence), while national origin only played a minor role [6]. Specifically related to nitrite-free salami, both genders were willing to pay more for nitrite-free salami, as are both sedentary and physically active consumers, with physically active consumers being willing to pay more. Meanwhile, age did not influence the willingness to pay an additional price with all investigated age groups (18–29; 30–39; ≥40), with a large degree being willing to pay more for nitrite-free salami [6]. As “organic” was not included as a factor in the Italian study, it is impossible to predict the importance of “organic” vs. “free from artificial additives/ingredients”.

Our market assessment established that organic, cured meat products are readily available on the Austrian market, whereas organic, uncured meat products are rare. However, based on consumer demands for clean label products, and, specifically, meat products free from additives and ingredients such as nitrite and nitrate, this is a market which is likely to grow in the near future. Producers will need to assess the consumer demands of the specific market and be aware of the potential effects of using natural additives in place of synthetic nitrite and nitrate on the quality and shelf life of the meat products. Our results have shown that it is indeed possible to produce meat products with natural curing ingredients (herbs and spices) that have comparable quality characteristics to their cured counterparts, although products containing neither synthetic nitrite/nitrate nor natural curing ingredients will appear less red due to the lack of formation of cured meat color.

## 4. Conclusions

Both pH and a_w_ were within the expected ranges with minor product-to-product variations that can be explained by differences in recipes and manufacturing. The microbial quality of the products was as expected for the product types, though with a higher total aerobic count and a slightly higher LAB count on day 0 for the uncured ham than for the cured ham samples. The uncured samples appeared to be less red (lower a*) than the cured samples, but the surface color did not change much during storage, though with a tendency for a* in salamis to be higher after storage in unopened packages than in open packages. Residual nitrite and nitrate were, not surprisingly, lowest in the uncured products, whereas the organic, cured products mostly contained similar amounts of residual nitrite and nitrate compared to conventional, cured products. Volatile N-nitrosamines may be formed during the production of cured meat products, during storage, or as a result of a potential second cooking step, as well as in the acidic environment of the stomach from ingested nitrite and nitrate. The detection of NDPhA in all investigated meat products at day 0 is a finding that warrants further investigation to be able to set forth recommendations for the meat processing industry and regulatory bodies. This could include reanalyzing the same products but from new batches, analyzing new products from the product groups already investigated, and analyzing other types of cured meat products with a special emphasis on comparing products of organic and conventional origin. Also, in alignment with current clean label trends, additional consumer-relevant storage times and conditions should be tested as well as further product groups. It would also be relevant to conduct a study of meat products cured with alternative curing agents in controlled amounts.

In conclusion, we found no evidence that organic meat products and meat products manufactured without the addition of nitrite or nitrate would present specific food quality issues to consumers. On the other hand, consuming only uncured meat products may not eliminate the intake of nitrite and nitrate due to many meat products containing natural sources of nitrate. Communication with consumers should reflect this fact.

## Figures and Tables

**Table 1:** Product information and sample ID of ham and salami products used in this study.

Product Type	Organic, Traditionally Cured Sample ID	Conventional, Traditionally Cured (Control) Sample ID	Organic, Uncured Sample ID
Salami	SOC	SCC	SOU
Cooked ham	HOC1	HCC	HOU
	HOC2	---	---

**Table 2:** Sampling plan and analyses conducted.

Storage	Analyses Day 0	Analyses Day 7
N/A	Nitrite Nitrate N-nitrosamines pH a_w_ Microbiology	---
Open (aerobic, 4 °C)	Color	Nitrite Nitrate N-nitrosamines Color pH a_w_ Microbiology
Unopened (oxygen-free, 4 °C)	---	Nitrite Nitrate N-nitrosamines Color pH a_w_ Microbiology

**Table 3:** Content of nitrite (NaNO_2_) and nitrate (KNO_3_); mean ± standard deviation (confidence intervals in square brackets) on day 0 and day 7 of open storage and day 7 unopened (n = 3, except for HOC1 where n = 1). When measurement was below limit of detection (LOD) for all triplicate samples, result is reported as <LOD. When measurement was <LOD for one or two replicas, value was set to 0.00 for calculation of average and standard deviation as well as for ANOVA, and average is marked with (*). Sample HOC1 was excluded from post hoc analysis due to lack of replicas. Different superscript letters indicate significant differences (*p* ≤ 0.05) within product type. Reference Table 1 for sample IDs.

Sample	Nitrite (mg/kg)			Nitrate (mg/kg)		
	Day 0	Day 7 Open	Day 7 Unopened	Day 0	Day 7 Open	Day 7 Unopened
Salami						
SOC	11.0 ^ab^ ± 0.7 [8.9–13.0]	12.1 ^ab^ ± 1.1 [8.8–15.4]	14.9 ^a^ ± 1.9 [9.0–20.8]	55.7 ^b^ ± 2.5 [48.1–63.4]	60.2 ^b^ ± 14.8 [15.2–105.1]	55.3 ^b^ ± 0.5 [53.7–57.0]
SCC	10.1 ^b^ ± 0.3 [9.4–10.9]	9.2 ^b^ ± 4.2 [0–22.0]	9.2 ^b^ ± 0.2 [8.6–9.8]	87.6 ^a^ ± 10.8 [56.6–118.6]	70.9 ^ab^ ± 10.3 [39.5–102.3]	68.7 ^ab^ ± 17.2 [16.2–121.1]
SOU	8.4 ^b^ ± 0.7 [6.3–10.4]	8.2 ^b^ ± 0.8 [5.7–10.6]	8.4 ^b^ ± 0.4 [7.1–9.7]	52.0 ^b^ ± 3.8 [40.4–63.6]	54.1 ^b^ ± 6.2 [35.3–72.9]	47.9 ^b^ ± 2.8 [39.5–56.2]
Cooked ham						
HOC1	11.8	13.0	10.7	28.4	33.5	24.6
HOC2	11.9 ^b^ ± 0.2 [11.2–12.5]	10.3 ^b^ ± 0.5 [8.7–11.9]	10.2 ^b^ ± 0.2 [9.6–10.8]	25.3 ^b^ ± 3.0 [16.3–34.3]	55.0 ^a^ ± 1.2 [51.3–58.8]	48.7 ^a^ ± 1.7 [43.7–53.8]
HCC	13.5 ^a^ ± 1.3 [9.5–17.6]	13.2 ^a^ ± 0.9 [10.5–15.9]	13.6 ^a^ ± 1.3 [9.6–17.6]	21.4 ^abc^ ± 1.0 [18.4–24.3]	22.2 ^bc^ ± 3.9 [10.3–34.1]	17.9 ^c^ ± 5.4 [1.5–34.2]
HOU	<LOD ^c^	<LOD ^c^	4.2 *^c^ ± 3.7 [3.2–9.5]	<LOD ^d^	<LOD ^d^	<LOD ^d^

**Table 4:** Content of N-nitrosamine N-nitrosodiphenylamine (NDPhA) on day 0 ± standard deviation (n = 3, except for HOC1 where n = 1) (confidence intervals in square brackets). NDPhA was not detected on day 7, and no other N-nitrosamines were detected in samples. Sample HOC1 was excluded from post hoc analysis due to lack of replicas. Different superscript letters signify significant differences (*p* ≤ 0.05) within product type. Reference Table 1 for sample IDs.

Sample	NDPhA (µg/kg)		
	Day 0	Day 7 Open	Day 7 Unopened
Salami			
SOC	31.5 ^a^ ± 10.1 [0.7–62.4]	ND	ND
SCC	12.9 ^b^ ± 3.7 [1.8–24.1]	ND	ND
SOU	10.2 ^b^ ± 1.2 [6.6–13.9]	ND	ND
Cooked ham			
HOC1	7.1	ND	ND
HOC2	6.6 ^b^ ± 0.8 [4.3–9.0]	ND	ND
HCC	169.6 ^a^ ± 93.5 [0–454.2]	ND	ND
HOU	45.1 ^ab^ ± 31.9 [0–142.0]	ND	ND

**Table 5:** Water activity (a_w_) and pH ± standard deviation (SD) for all samples on day 0 and day 7 (n = 3 for each storage time/condition, except for HOC1 where n = 1) (confidence intervals in square brackets). Different superscript letters indicate significant differences (*p* ≤ 0.05). Reference Table 1 for sample IDs.

Sample	a_w_			pH		
	Day 0	Day 7 Open	Day 7 Unopened	Day 0	Day 7 Open	Day 7 Unopened
Salami						
SOC	0.812 ^ac^ ± 0.012 [0.777–0.848]	0.776 ^b^ ± 0.016 [0.728–0.825]	0.816 ^ac^ ± 0.021 [0.751–0.880]	5.59 ^a^ ± 0.11 [5.26–5.93]	5.46 ^b^ ± 0.11 [5.13–5.78]	5.68 ^a^ ± 0.07 [5.48–5.88]
SCC	0.843 ^d^ ± 0.006 [0.824–0.862]	0.812 ^a^ ± 0.003 [0.804–0.820]	0.845 ^d^ ± 0.013 [0.805–0.885]	5.33 ^bc^ ± 0.03 [5.23–5.42]	5.27 ^cd^ ± 0.01 [5.25–5.28]	5.24 ^cd^ ± 0.02 [5.20–5.29]
SOU	0.833 ^cd^ ± 0.005 [0.819–0.847]	0.818 ^ac^ ± 0.014 [0.774–0.861]	0.825 ^d^ ± 0.015 [0.779–0.871]	5.18 ^d^ ± 0.04 [5.07–5.29]	5.26 ^cd^ ± 0.13 [4.86–5.65]	5.18 ^d^ ± 0.10 [4.88–5.47]
Cooked ham						
HOC1	0.943	0.940	0.938	6.27	6.38	6.41
HOC2	0.953 ^a^ ± 0.002 [0.948–0.958]	0.939 ^b^ ± 0.002 [0.932–0.945]	0.940 ^bc^ ± 0.002 [0.933–0.946]	6.32 ^abc^ ± 0.12 [5.96–6.68]	6.10 ^aef^ ± 0.12 [5.73–6.47]	6.18 ^abf^ ± 0.12 [5.83–6.53]
HCC	0.940 ^bc^ ± 0.006 [0.923–0.956]	0.941 ^bcd^ ± 0.005 [0.927–0.954]	0.937 ^b^ ± 0.001 [0.934–0.941]	6.54 ^d^ ± 0.08 [6.30–6.79]	6.37 ^c^ ± 0.10 [6.08–6.66]	6.33 ^bc^ ± 0.06 [6.17–6.50]
HOU	0.945 ^e^ ± 0.002 [0.939–0.952]	0.947 ^de^ ± 0.003 [0.939–0.955]	0.944 ^cde^ ± 0.001 [0.941–0.947]	6.24 ^abf^ ± 0.27 [5.41–7.08]	5.94 ^e^ ± 0.14 [5.52–6.36]	6.05 ^ef^ ± 0.04 [5.95–6.16]

**Table 6:** Surface color for all samples on day 0 and day 7 after storage in opened packages, and day 7 after storage in unopened packages (control) (n = 3 except for HOC1 where n = 1) (confidence intervals in square brackets). Reference Table 1 for sample IDs. Within rows, different superscript letters indicate significant differences (*p* ≤ 0.05) for each color variable L*, a*, and b* during open storage for seven days (a–b) or between open and unopened storage on day 7 (x–y).

Sample	Day 0			Day 7 Open			Day 7 Unopened		
	L*	a*	b*	L*	a*	b*	L*	a*	b*
Salami									
SOC	43.76 ± 2.90 [34.94–52.57]	14.86 ± 1.93 [8.99–20.72]	9.86 ± 1.26 [6.04–13.69]	44.55 ± 3.52 [33.82–55.28]	15.65 ± 2.93 [6.74–24.57]	9.12 ± 2.97 [0.10–18.15]	46.40 ± 2.12 [39.94–52.85]	16.26 ± 1.69 [11.13–21.38]	9.12 ± 1.70 [3.94–14.30
SCC	46.24 ± 1.73 [40.99–51.50]	17.00 ± 1.80 [11.51–22.48]	12.06 ± 2.73 [4.83–19.28]	46.13 ± 3.01 [36.97–55.39]	17.77 ^y^ ± 2.08 [11.43–24.10]	11.54 ± 2.26 [4.98–18.11]	46.67 ± 2.83 [38.06–55.28]	19.75 ^x^ ± 1.12 [16.34–23.15]	11.94 ± 2.11 [5.54–18.35]
SOU	40.32 ± 1.92 [34.43–46.16]	10.49 ± 1.41 [6.20–14.77]	6.41 ± 1.07 [3.15–9.97]	41.16 ± 4.84 [26.43–55.89]	10.30 ^y^ ± 2.50 [2.71–17.90]	5.09 ± 2.18 [−1.55–11.73]	40.68 ± 2.42 [33.32–48.04]	12.44 ^x^ ± 1.62 [7.51–17.36]	6.19 ± 1.09 [2.89–9.50]
Cooked ham									
HOC1	66.20	11.12	6.91	60.80	10.10	7.29	58.49	10.72	8.34
HOC2	71.90 ± 2.07 [65.59–78.21]	9.59 ± 1.41 [5.30–13.88]	8.58 ± 1.02 [5.48–11.69]	64.78 ± 2.82 [56.20–73.36]	9.61 ± 1.31 [5.62–13.61]	9.06 ^x^ ± 0.48 [7.61–10.51]	66.01 ± 2.29 [59.05–72.96]	9.17 ± 1.63 [4.21–14.13]	7.74 ^y^ ± 0.77 [5.40–10.09]
HCC	65.04 ^a^ ± 2.33 [57.94–72.13]	9.90 ± 1.70 [4.74–15.07]	6.43 ^a^ ± 1.33 [2.36–10.49]	58.13 ^b^ ± 2.45 [50.67–65.59]	9.09 ± 0.98 [6.12–12.06]	5.61 ^b^ ± 0.77 [3.27–7.95]	60.03 ± 2.46 [52.55–67.52]	8.33 ± 2.21 [1.60–15.07]	5.77 ± 1.64 [0.77—10.77]
HOU	76.20 ± 1.05 [73.00–79.41]	1.46 ± 0.64 [−0.59–3.51]	8.82 ^b^ ± 0.89 [6.11–11.55]	75.89 ± 1.98 [69.87–81.91]	1.11 ± 0.36 [0.02–2.20]	10.04 ^ax^ ± 0.70 [7.91–12.17]	77.01 ± 2.41 [69.68–84.34]	1.35 ± 0.75 [−0.94–3.65]	8.11 ^y^ ± 0.57 [6.37–9.85]

## Data Availability

The data presented in this study are available on request from the corresponding author. The data are not publicly available due to privacy restrictions.
